# The Internal Secretions and Principles of Medicine

**Published:** 1904-03

**Authors:** 


The Internal Secretions and Principles of Medicine. By Charles E. de M. Sajous, M. D., of Philadelphia; Fellow of the College of Physicians of Philadelphia; Member of the American Philosophical Society and Academy of Natural Science, etc. Volume I. With forty-two illustrations. Pages, 800.

This is an advance work ahead of the times. It is based upon experiment, where permanent volumes are obtained. A study of the adrenals, as viewed from clinical pathology, occupies the first sixty-two pages. The next 100 pages are devoted to the internal secretions of the adrenals in its relations to the respiratory processes and the composition of the blood.

The third chapter consists in internal secretion of the adrenals in its relations to the general oxidation processes. Chapter four takes up the thyroid and thymus glands in their relations to the adrenals. The anterior pituitary body, the thyroid gland and the adrenals, as parts of an autonomous system, are discussed in chapter five. The other seven chapters are equally as interesting and instructive as the ones mentioned. The work, as a whole, is one of the most thoroughly scientific and advanced of the last half dozen years. T. J. B.

